# Insertion of an endogenous Jaagsiekte sheep retrovirus element into the BCO2 - gene abolishes its function and leads to yellow discoloration of adipose tissue in Norwegian Spælsau (*Ovis aries*)

**DOI:** 10.1186/s12864-021-07826-5

**Published:** 2021-06-30

**Authors:** Matthew Kent, Michel Moser, Inger Anne Boman, Kristine Lindtveit, Mariann Árnyasi, Kristil Kindem Sundsaasen, Dag Inge Våge

**Affiliations:** 1 Department of Animal and Aquacultural Sciences, Centre for Integrative Genetics (CIGENE), Faculty of Biosciences, Norwegian University of Life Sciences, No-1432 Ås, Norway; 2 The Norwegian Association of Sheep and Goat Breeders, No-1431 Ås, Norway

**Keywords:** Sheep, spælsau, *BCO2*, Structural variant, Nanopore sequencing, Functional mutation, Yellow fat, Endogenous Jaagsiekte sheep retrovirus

## Abstract

**Background:**

The accumulation of carotenoids in adipose tissue leading to yellow fat is, in sheep, a heritable recessive trait that can be attributed to a nonsense mutation in the *beta-carotene oxygenase 2 (BCO2)* gene. However, not all sheep breeds suffering from yellow fat have this nonsense mutation, meaning that other functional mechanisms must exist. We investigated one such breed, the Norwegian spælsau.

**Results:**

In spælsau we detected an aberration in *BCO2* mRNA. Nanopore sequencing of genomic DNA revealed the insertion of a 7.9 kb endogenous Jaagsiekte Sheep Retrovirus (enJSRV) sequence in the first intron of the *BCO2* gene. Close examination of its cDNA revealed that the *BCO2* genes first exon was spliced together with enJSRV-sequence immediately downstream of a potential -AG splice acceptor site at enJSRV position 415. The hybrid protein product consists of 29 amino acids coded by the *BCO2* exon 1, one amino acid coded by the junction sequence, followed by 28 amino acids arbitrary coded for by the enJSRV-sequence, before a translation stop codon is reached.

**Conclusions:**

Considering that the functional BCO2 protein consists of 575 amino acids, it is unlikely that the 58 amino acid BCO2/enJSRV hybrid protein can display any enzymatic function. The existence of this novel *BCO2* allele represents an alternative functional mechanism accounting for *BCO2* inactivation and is a perfect example of the potential benefits for searching for structural variants using long-read sequencing data.

**Supplementary Information:**

The online version contains supplementary material available at 10.1186/s12864-021-07826-5.

## Background

The yellow coloration of adipose tissue in sheep is known to be a heritable trait in different sheep breeds and is caused by the accumulation of carotenoids [1–4]. In terms of consumer preferences, “yellow fat” is an undesirable meat quality that leads to loss of product value and is therefore not wanted in meat animals.

In animals, two enzymes displaying different cleavage effects on carotenoids have been identified; *BCO1* (*beta-carotene oxygenase 1*) which cleaves β-carotene symmetrically into two retinal molecules, and *BCO2* (*beta-carotene oxygenase 2*) which cleaves β-carotene asymmetrically into β-apo-10′-carotenal (C27) and β-ionone (C13) [5–8]. An important function of these two enzymes is to execute the first step in degradation of the large amounts of carotenoids absorbed from the plant rich diet of ruminants. Functional failure of these enzymes may lead to accumulation of intact carotenoids in body tissues like fat or muscle. This effect has been illustrated by mutations influencing either transcript levels or protein coding sequence for both genes in several organisms [9–12]. Mutations in *BCO2* especially seem to result in accumulation of carotenoids and yellow colouration of tissues [13, 14], possibly due to its broader substrate specificity [15, 16].

Earlier we have reported that a mutation that introduces a stop codon in amino acid position 66 of ovine *BCO2* is associated with yellow fat in Norwegian White Sheep breed [12]. However, in the native Norwegian breed Spælsau, the yellow fat trait is found to segregate in the absence of this mutation, suggesting that alternative functional mechanism is influencing this trait. In the present study we searched for novel *BCO2* mutations in the Spælsau breed that could explain the yellow fat phenotype and included Nanopore long range sequencing to also detect structural variants that possibly could influence this trait.

## Results

**Fig. 1:**
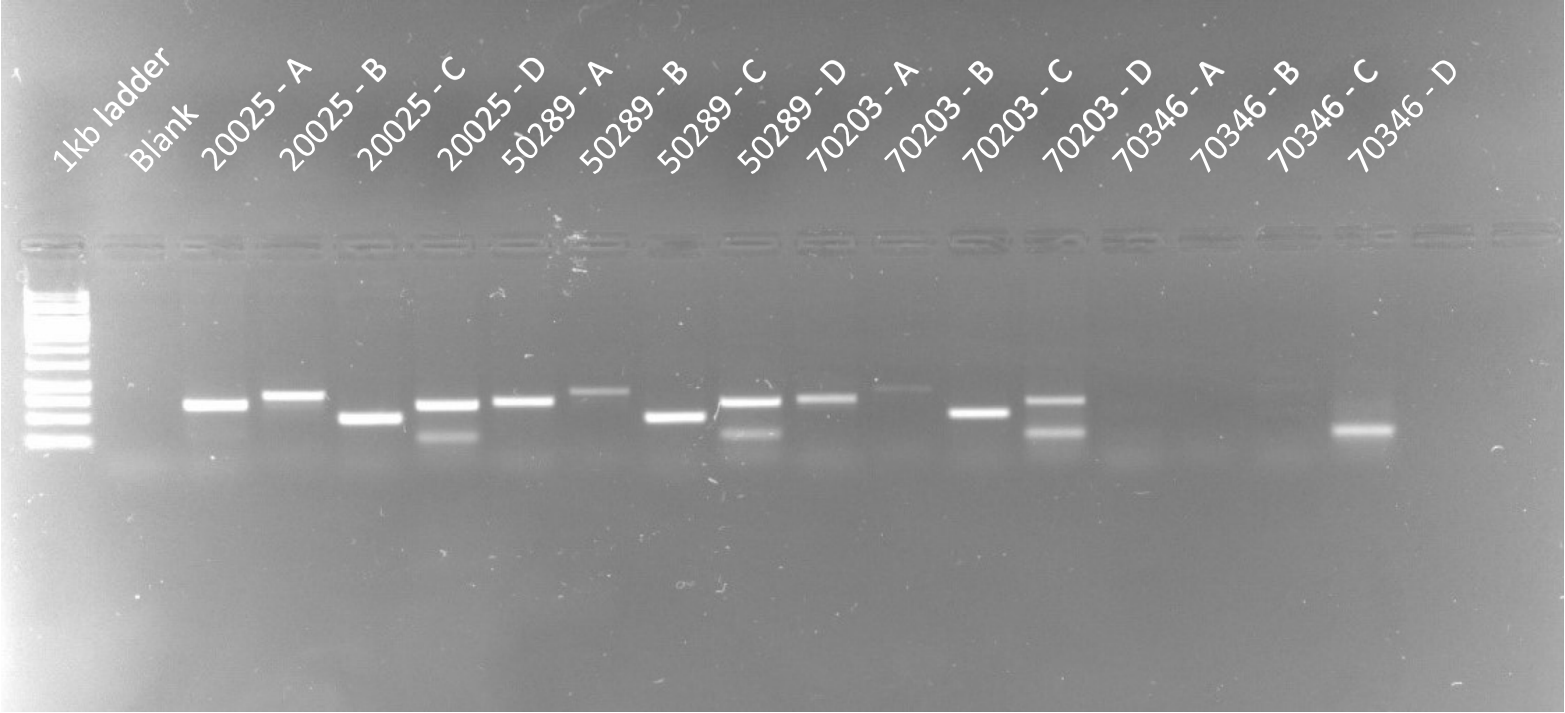
Figure shows 4 different sets of primers (A: 7541–7542, B: 7543–7544, C: 7545–7546, D: 7547–7548) amplifying different subregions of the ovine BCO2 cDNA sequence in 4 different individuals (20025, 50289, 70203 and 70346, respectively). No fragments are amplified in the yellow fat individual (70346). The band in 70346 - D is an artefact which also is visible in the 3 other individuals, in addition to the band of expected size found in the other 3. The full-length, unprocessed gel picture is found in Fig. S2a. The size marker is 1 kb GeneRuler®

Our initial experiments (sample set 1) to better understand the mechanism(s) responsible for yellow fat in Norwegian Spælsau included performing PCR on cDNA from animals presenting white or yellow fat to detect expression of *BCO2*. Four primer pairs targeting *BCO2* produced 4 fragments of expected size in the three individuals showing white fat, while no fragments were amplified from the individual with yellow fat (Fig. 1).

Genomic DNA from the single yellow fat individual (70346) and from an individual with the white fat phenotype (20025) was sequenced using nanopore long read technology. Over the course of two consecutive PromethION sequencing runs, one flow-cell yielded 6,908,462 reads (73 Gb raw data). After demultiplexing, 78% of reads (*n* = 5,387,322) were assigned either to sample 20025 (1,330,100 reads; 21 Gb) or to sample 70346 (2,512,544 reads; 27.2 Gb). After filtering for quality and read length (Q > 7, length > 4 kb) a total of 20.2 Gb data remained for individual 20025 (≈7X coverage; median read length = 14,792, N50 read length = 23,141) and 25.8 Gb for individual 70346 (≈9X coverage, median read length = 10,567, N50 read length = 13,570 bp).

**Fig. 2:**
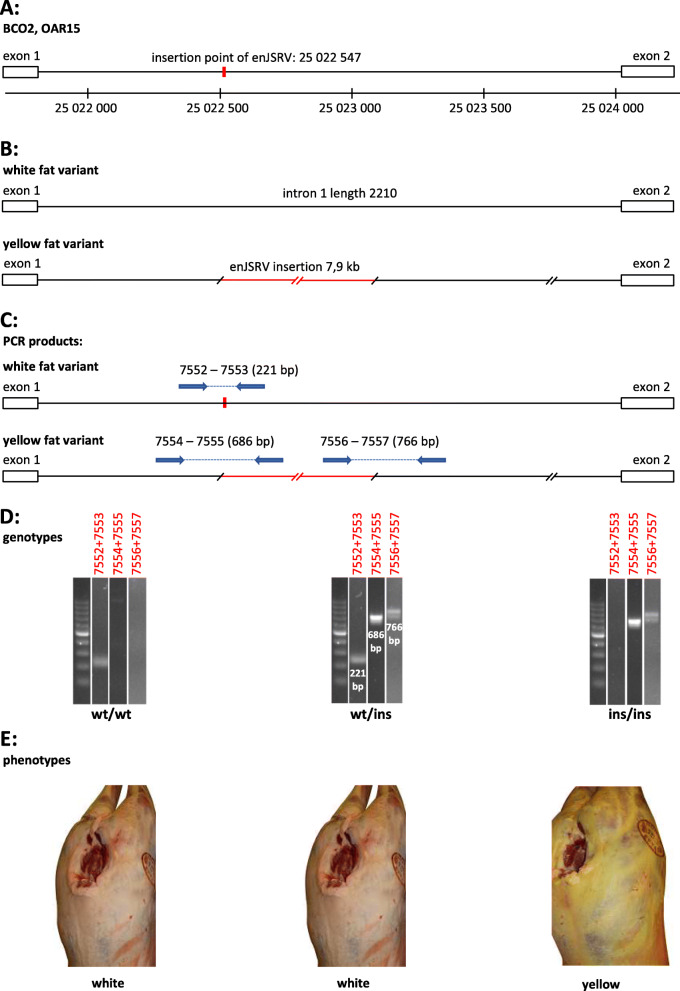
A 7.9 kb endogenous Jaagsiekte Sheep Retrovirus (enJSRV) element inserted into the first intron of the ovine *BCO2 -* gene. **A** The *BCO2* first intron is shown together with exon 1 and exon 2. The insertion point of the enJSRV is indicated in red, 730 bp downstream of exon 1. The line below is showing the corresponding chromosome 15 positions in bp. **B** *BCO2* intron 1 is shown with and without the inserted endogenous virus element. **C** Primer pairs for amplifying the enJSRV insertion point when no insertion is present (7552/7553), the upstream intron 1/enJSRV junction (7554/7555) and the downstream enJSRV/intron 1 juction (7556/7557). Distances are not drawn to scale. **D** Gel separations of PCR-products resulting from the primer combinations shown in the previous section (c). Genotypes are scored as wild type (wt) or carrier of the endogenous Jaagsiekte Sheep Retrovirus (ins). Example lanes are copied from 3 different gels used for genotyping (Fig. S2b,c,d). The primer combinations 7552/7553, 7554/7555 and 7556/7557 are shown from left to right. The lanes resulting from each of the 3 primer pairs are grouped together for the genotypes wt/wt, wt/ins and ins/ins, respectively. The size marker in lane 1 is 100 bp GeneRuler®. **E** The corresponding phenotypes of the genotypes shown in previous section (d). Photo: Nortura SA

Filtered nanopore reads were mapped to the Oar_rambouillet_v1.0 reference and examined for SV’s. Within the 70 kb genomic interval harbouring *BCO2* (NC_040266.1: 25,021,687-25,091,194), 7 SV’s were detected. Six of these (see Table S1) were found in both individuals (both white and yellow fat phenotype). These were disregarded as candidate SVs explaining the yellow fat phenotype. The single remaining SV consisted of a 7939 bp insertion at reference genome position NC_040266.1: 25,022,547 which is 730 bp downstream from the end of *BCO2*’s first exon (Fig. 2). The inserted sequence showed high similarity (99.0% identity) and covered the full length (7941 bp) of the endogenous Jaagsiekte sheep retrovirus (enJSRV; accession MF175071.1). Compared to the virus genome reference sequence (NC_001494.1), which is 7462 bp long, the endogenous version is 479 bp longer due to longer repeats at each end of the enJSRV. The virus sequence is 92.8% identical and shows 98% coverage to the inserted enJSRV sequence. The enJSRV insertion was verified with PCR using primer pairs 7554–7555 and 7556–7557 in the two sequenced animals (20025 and 70346), which gave expected products of 686 bp and 766 bp, respectively, spanning the upstream and downstream sheep *BCO2* - enJSRV junctions, respectively. The primer pair spanning the insertions site 7552–7553 did not amplify any product in the yellow fat animal (70346), while it did in the heterozygous white fat animal (20225) (Fig. 2). Complete *BCO2* intron 1 sequence, including the 7939 bp enJSRV insertion is available under accession LR701838.1.

To test whether exon 1 (located upstream of the insertion point) could still be expressed in a homozygous carrier we amplified a subregion of exon 1 (primers 7550–7551) from cDNA. A band of expected size (112 bp) was produced, indicating that *BCO2* exon 1 was being transcribed in individual 70346.

**Fig. 3:**
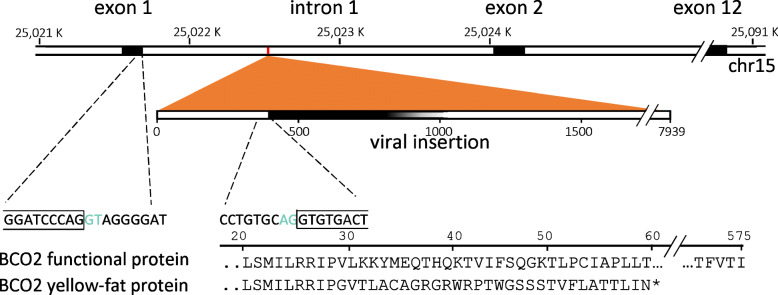
Diagram of the viral insertion in the *BCO2* gene (NC_040266.1: 25021687–25,091,194). The splice donor and acceptor sites are indicated in green. The solid dark colour in the viral insertion is indicating the protein translation start point in the spliced mRNA

By combining a *BCO2* exon 1 forward primer (7550) with enJSRV specific reverse primer (7555) we were able to generate a 188 bp PCR product using cDNA from the yellow fat individual (70346). Sanger sequencing this fragment between the two primer binding sites revealed it to be composed of 94 bp from *BCO2* exon 1, and 55 bp of enJSRV sequence (in a non-protein coding region), beginning at the base corresponding to position 415 in the enJSRV sequence (MF175071.1) (Fig. 3).

Analysis of sample set 2 (26 individuals with an expected relatively high frequency of the yellow fat allele) showed that 10 were heterozygous for the insertion, 4 were homozygous for the insertion, and the remaining 12 lambs were homozygous wild type. The abattoir’s meat-grading report noted that the 4 homozygous animals displayed yellow fat, while the remaining group had white fat (Table 1).

**Table 1:** Fat colour grading and genotypes of 26 lambs descending from two different rams (76245736 and 89012427) known to produce yellow fat offspring. The ewes also have an increased probability of carrying the yellow fat allele, due to relationship to known carriers. Genotypes are scored as wild type (wt) or carrier of the endogenous Jaagsiekte Sheep Retrovirus (ins) based on the PCR-test described in materials and methods

Lamb ID	Ram	Ewe	Sex	Phenotype	Genotype
90027	76245736	37200184	male	white fat	wt/ins
90029	76245736	37200184	male	white fat	wt/wt
90041	76245736	89740216	male	white fat	wt/ins
90059	76245736	37198929	female	white fat	wt/ins
90060	76245736	37198929	male	white fat	wt/wt
90072	76245736	89740213	female	white fat	wt/ins
90073	76245736	89740213	female	white fat	wt/wt
90001	89012427	82424293	female	**yellow fat**	**ins/ins**
90002	89012427	82424293	female	**yellow fat**	**ins/ins**
90015	89012427	76245888	female	white fat	wt/ins
90019	89012427	88844575	female	**yellow fat**	**ins/ins**
90075	89012427	76382061	female	white fat	wt/ins
90077	89012427	76382061	female	white fat	wt/wt
90083	89012427	37199669	female	white fat	wt/wt
90104	89012427	70456589	female	white fat	wt/ins
90115	89012427	82991541	female	white fat	wt/wt
90201	89012427	88842546	male	white fat	wt/wt
90202	89012427	88842546	male	white fat	wt/wt
90214	89012427	76245888	male	white fat	wt/wt
90228	89012427	76245885	male	white fat	wt/ins
90230	89012427	76245885	male	white fat	wt/wt
90297	89012427	89400436	male	**yellow fat**	**ins/ins**
90298	89012427	89400436	male	white fat	wt/wt
90307	89012427	37199669	male	white fat	wt/ins
90330	89012427	70456589	male	white fat	wt/wt
90336	89012427	82991541	male	white fat	wt/ins

## Discussion

In this study we investigated whether changes the *BCO2* gene could be responsible for yellow fat phenotype observed in Norwegian spælsau. By performing PCR on cDNA, we were able to confirm the expression of *BCO2* in 3 individuals displaying the white fat phenotype, however no amplification products were observed in the yellow fat individual. This indicated that the yellow fat phenotype was due to downregulated expression of *BCO2* mRNA, but our results did not offer insight into the mechanism underlying this.

To investigate if novel genomic rearrangements (for example structural variants) in the BCO2 region could account for the lack of detectable mRNA, we sequenced DNA from both a single yellow- and white-fat individual. This revealed the presence of a complete enJSRV-sequence inserted between exon 1 and exon 2 in the *BCO2* gene. By amplifying a subregion of exon 1 from cDNA, we could establish that the insertion does not appear to interfere with expression of exon 1 in a homozygous carrier, leading us to hypothesize that gene regulation is unaffected by the insertion and that the enJSRV sequence within intron 1 is interrupting the normal splicing process.

By combining a forward primer located in the *BCO2* exon 1 with a reverse primer located 470 bp into the enJSRV sequence, a hybrid cDNA product was amplified consisting of 94 bp from *BCO2* exon 1 and 55 bp of enJSRV sequence. If translated, this hybrid mRNA sequence would give rise to 29 amino acids of the wild-type ovine BCO2 protein chimerized to 1 amino acid coded by the junction sequence followed by 28 amino acids coded by the enJSRV-sequence, before the protein is terminated by a stop codon. Knowing that the full-length protein consists of 575 amino acids, we theorize that it is highly unlikely that these 58 peptides can perform the functions of wildtype BCO2 enzyme in animals homozygous for the enJSRV insertion.

To strengthen our finding that the enJSRV insertion is functionally impacting transcript processing of *BCO2*, we genotyped 26 additional individuals (sample set 2) with an expected high frequency of the yellow fat allele. Comparing our genotypes with the reported phenotypes of these 26 individuals, we observed a genotype pattern consistent with recessive inheritance of the yellow fat phenotype.

Due to the relatively low frequency of this phenotype in the population and access to individuals, the number of individuals included in the present study is low. However, based on the available evidence we conclude that the enJSRV insertion in *BCO2* (intron 1) is a strong candidate for the yellow fat phenotype in spæl sheep. To the best of our knowledge, this insertion also represents the first example of a structural variant (excluding copy number variation) affecting a quality trait in this production livestock species.

## Conclusions

In this study we identified an insertion of an endogenous Jaagsiekte Sheep Retrovirus element in the *BCO2* gene in the Norwegian spælsau breed by using Nanopore sequencing technology. The insertion is localised in the first intron of *BCO2* and interrupts the mRNA splicing process. The resulting mRNA consists of *BCO2* exon 1 sequence fused with endogenous retrovirus sequence, leaving an open reading frame of totally 58 amino acids. Given that the wild type BCO2 enzyme consists of 575 amino acids, we find it highly unlikely that the hybrid protein of 58 amino acids can have any enzyme function. Our results make it possible for the sheep farming industry to screen their breeding candidates for this variant to avoid the yellow fat phenotype. We also think this paper clearly illustrate the strength of using long-read sequencing technology to search for structural variation and it also exemplifies how such variation can directly influence gene function.

## Methods

### Animals

In Norwegian abattoirs, fat colour in sheep carcasses is graded as “normal” (white) or “yellow” and recorded at the individual level within the Norwegian Sheep Recording System. For sample set 1, we obtained samples from 4 Spælsau animals at slaughter from a private farm with a history of producing yellow fat lambs (sample set 1). Liver samples (approximately 0.125 cm^3^) from a yellow fat male (individual 70346), and from two white fat males (50289,70203) and one white fat female (20025) were collected and stored in RNAlater™ (QIAGEN, Hilden, Germany). The white fat individuals may be carriers of the genetic variant(s) causing yellow fat, based on their relationship to known yellow fat animals, but most likely not homozygous.

Subsequently, a larger set of 26 Spælsau lamb samples (Table 1) were collected from two private farms in Norway (sample set 2). Lambs from each farm were half-sibs descending from one of two rams registered with some offspring displaying the yellow fat trait. Similarly, mothers of these lambs were also known to be related to individuals presenting yellow fat, and therefore had an increased probability of carrying at least one yellow fat allele. To be specific, 5 ewes had offspring with yellow fat, 9 ewes had a parent with offspring with yellow fat and one ewe was half-sib of individual 70203 in sample set 1. The farms contributing to both sample set 1 and 2 are members of the Norwegian Sheep Recording System.

### RNA extraction and cDNA synthesis

RNA was extracted using the RNeasy®Plus Universal Mini Kit from QIAGEN according to the manufacturer’s instructions. The concentration and purity of the RNA was measured using a NanoDrop 8000 (Thermo Scientific), and the integrity of RNA was measured using a Bioanalyzer 2100 (Agilent). All samples had an RNA integrity value (RIN) of at least 8.1. cDNA was produced using a SuperScript™ II Reverse Transcriptase kit from Invitrogen according to manufacturer’s instructions.

### PCR amplification of BCO2 cDNA

To amplify the complete cDNA sequence of *BCO2*, four pairs of validated primers (A: 7541–7542, B: 7543–7544, C: 7545–7546, D:7547–7548) were mixed with cDNA and AmpliTaq Gold® Polymerase (Applied Biosystems) in four separate PCR reactions generating overlapping regions of the *BCO2* cDNA [12]. The PCR was performed using 10 min at 95 °C, and 40 cycles of 95 °C for 30 s, 57 °C for 30 s and 72 °C for 1.5 min. Expected fragment sizes are 692 bp, 822 bp, 491 bp and 660 bp for primer pairs A, B, C and D, respectively (Fig. 1).

### Nanopore sequencing

High-molecular-weight DNA from white (20025) and yellow fat (70346) individuals was extracted from liver tissue using Genomic-tips (G/100) from Qiagen, and fragments smaller than 25Kb were progressively depleted using a Short Read Eliminator kit (Circulomics; USA). Sequencing libraries were prepared using Ligation sequencing kit (LSK109, Oxford Nanopore) with native barcoding (EXP-NBD104, Oxford Nanopore). Equal masses of libraries were combined, 300 ng was loaded into a single PromethION flowcell (FLO-PRO002). After 23 h the run was stopped (producing 36 Gb raw data) and the flow-cell regenerated according to Oxford Nanopore’s nuclease flush protocol. The remaining pooled library (230 ng) was added to the same flow-cell and a new sequencing run performed (22.2 h; 22 Gb raw data).

### Base-calling and quality filtering

Nanopore reads were base-called using Guppy, version 3.0.3 (Oxford Nanopore, MinKNOW v.19.05.1) using the ‘High accuracy’ flip-flop model. Reads from the two sequencing runs were merged and demultiplexed with QCAT (https://github.com/nanoporetech/qcat). Demultiplexed reads from each sample were trimmed for 50 bp from the start and quality-filtered for average PHRED quality > 7 and minimum length of 4000 bp with fastp (v0.19.5) options “ –disable trim_poly_g --disable_adapter_trimming -q 7 -l 4000 -f 50 “ [17].

### Structural variant detection

Quality-filtered reads were mapped to the sheep reference genome Oar_rambouillet_v1.0 (RefSeq accession GCF_002742125.1) using minimap2 (v2.16-r922) [18]. Structural variants (SV’s) were called with the SV-caller SVIM (v0.5.0) using default parameters [19]. SVs within 100 Kbp of the candidate gene *BCO2* (NM_001159278.1) were inspected visually with Integrative Genomics Viewer (IGV) [20].

As a complementary approach to identify larger rearrangements between the sheep reference genome and the yellow fat individual (70346), quality-filtered reads of individual 70346 were assembled using Flye software (v2.4.2) with options “--genome-size 2.8g -m 10000” [21]. Contigs from the individual 70346 genome assembly were mapped to the sheep reference genome using minimap2 (v2.16-r922) [18]. Candidate contigs spanning *BCO2*-region were aligned and dot-plotted against the sheep reference genome using Gepard (v1.40) for visual inspection [22], (see Fig. S1).

### Identification of intronic insertion in BCO2

Candidate SVs within the *BCO2* gene region were filtered for presence only in individual 70346 or following expected allele pattern among the two individuals (homozygous in individual 70346 and heterozygous in individual 20025. On-line blastn [23] was used to identify the inserted sequence in *BCO2* intronic region (INS_chr15_25022547). Insertion boundaries were determined by aligning contigs of sheep_70346 genome assembly against the Oar_rambouillet_v1.0 reference genome.

### Detection of BCO2 exon 1 and BCO2- JSRV hybrid cDNA in the yellow fat animal

A 112 bp exon 1 fragment of *BCO2* from the yellow fat individual (70346) was amplified using primers 7550/7551 (Table 2). A hybrid fragment consisting of sheep *BCO2* exon 1 sequence and JSRV sequence were amplified from the same individual using primers 7550/7555. In both cases cDNA was used as template with the following thermo cycler program: 10 min at 95 °C and 40 cycles at 95 °C for 30 s, 57 °C for 30 s and 72 °C for 30 s using AmpliTaq Gold® Polymerase (Applied Biosystems). The 7550/7555 product was DNA sequenced by Sanger technology at Eurofins Scientific, Luxembourg, using the same two primers for the sequencing reaction.

**Table 2:** Primers used in addition to those described in Våge & Boman 2010

Primer	Sequence 5′ - 3’	Position	Direction	NCBI reference seq
7550	CTGCTGCTGCAGAACTCAAC	24–43	F	NM_001159278
7551	GGGATCCGGCGTAATATCAT	116–135	R	NM_001159278
7552	AATCCCATGGACGGAGAAG	25,022,430–25,022,448	F	NC_040266.1
7553	CTCCCTTTAAGAGGCAGCAT	25,022,631–25,022,650	R	NC_040266.1
7554	CAGGAAACCTGGGTTCGAT	530–548	F	LR701838.1
7555	GGCGAGGAAAACTGTCGAG	1197–1215	R	LR701838.1
7556	CACCAACATCTTATGGAGCTTTT	8182–8204	F	LR701838.1
7557	GGGTTCTTTGCCATTAGCAC	8928–8947	R	LR701838.1

### PCR to detect the presence or absence of the endogenous virus sequence in genomic DNA

To detect the presence or absence of endogenous virus sequence in genomic DNA, three pairs of primers were designed (Table 2): one pair (7552/7553) was designed to amplify across the insertion point without any insertion, one pair (7554/7555) to amplify the junction between the *BCO2* intron sequence and the upstream enJSRV region and one (7556/7557) to amplify the junction between the *BCO2* intron sequence and the downstream enJSRV region. Expected fragments lengths are 221 bp, 686 bp and 766 bp, respectively. DNA was denatured for 10 min at 95 °C and PCR run for 40 cycles at 95 °C for 30 s, 58 °C for 30 s and 72 °C for 1.5 min using using AmpliTaq Gold® Polymerase (Applied Biosystems).

## Supplementary Information

**Additional file 1: Table S1.** Contain information about 6 additional structural variants detected within the 70 kb genomic interval harbouring BCO2 gene.

**Additional file 2: Figure S1.** A plot showing the alignment of a the 70 kb contig constructed from nanopore reads from a yellow-fat individual aligned to the ovine reference genome

**Additional file 3: Figure S2.** Full-length, unprocessed gel pictures of the gels shown in Fig. 1 and Fig. 2D.

## Data Availability

Raw long-range sequencing data have been submitted to the European Nucleotide Archive (https://www.ebi.ac.uk/ena/browser/home) under accession number ERR3522460 and ERR3522461. Complete *BCO2* intron 1 sequence, including the 7939 bp enJSRV insertion is available under European Nucleotide Archive accession LR701838.1. The hybrid mRNA sequence is available under GenBank (https://www.ncbi.nlm.nih.gov/genbank/) accession number MT024238.

## Abbrevations

BCO1: beta-carotene oxygenase 1.
BCO2: beta-carotene oxygenase 2.
enJSRV: endogenous Jaagsiekte Sheep Retrovirus.
