# Supplementary material for: Insertion of an endogenous Jaagsiekte sheep retrovirus element into the BCO2 - gene abolishes its function and leads to yellow discoloration of adipose tissue in Norwegian Spælsau (Ovis aries)
Source: BMC Genomics. 2021 Jun 30;22:492. doi: 10.1186/s12864-021-07826-5 (PMC8247158; doi:10.1186/s12864-021-07826-5)
Supplement: Supplementary file 1 — Additional file 1: Table S1. Contain information about 6 additional structural variants detected within the 70 kb genomic interval harbouring BCO2 gene. [file 12864_2021_7826_MOESM1_ESM.docx]

Supplementary Table 1:

| Ref Sequence | Position | SV relative to reference | SV length |
| --- | --- | --- | --- |
| NC_040266.1 | 25035406 | Novel insertion | 178 |
| NC_040266.1 | 25051293 | Novel insertion | 156 |
| NC_040266.1 | 25074561 | Deletion | 151 |
| NC_040266.1 | 25077920 | Deletion | 867 |
| NC_040266.1 | 25077931 | Deletion | 1657 |
| NC_040266.1 | 25078797 | Deletion | 790 |

Table listing positions and sizes of the 6 additional structural variants detected within the 70kb genomic interval harbouring BCO2 (NC_040266.1: 25,021,687-25,091,194).
