# Supplementary material for: Insertion of an endogenous Jaagsiekte sheep retrovirus element into the BCO2 - gene abolishes its function and leads to yellow discoloration of adipose tissue in Norwegian Spælsau (Ovis aries)
Source: BMC Genomics. 2021 Jun 30;22:492. doi: 10.1186/s12864-021-07826-5 (PMC8247158; doi:10.1186/s12864-021-07826-5)
Supplement: Supplementary file 2 — Additional file 2: Figure S1. A plot showing the alignment of a the 70 kb contig constructed from nanopore reads from a yellow-fat individual aligned to the ovine reference genome [file 12864_2021_7826_MOESM2_ESM.docx]

Supplementary Figure 1:


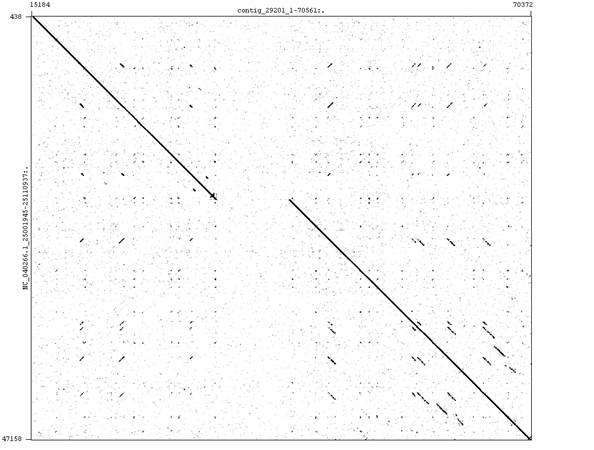


Alignment of a 70,561bp contig constructed from nanopore reads from a yellow-fat individual (70346; x-axis) aligned to the ovine reference genome (NC_040266.1: 25,001,945-25,110,937; y-axis). The break in alignment is attributed to the insertion of 7939bp sequence in the 70346 contig.
