# Supplementary material for: Insertion of an endogenous Jaagsiekte sheep retrovirus element into the BCO2 - gene abolishes its function and leads to yellow discoloration of adipose tissue in Norwegian Spælsau (Ovis aries)
Source: BMC Genomics. 2021 Jun 30;22:492. doi: 10.1186/s12864-021-07826-5 (PMC8247158; doi:10.1186/s12864-021-07826-5)
Supplement: Supplementary file 3 — Additional file 3: Figure S2. Full-length, unprocessed gel pictures of the gels shown in Fig. 1 and Fig. 2D. [file 12864_2021_7826_MOESM3_ESM.docx]

Supplementary Figure 2:

*Figure S2a*


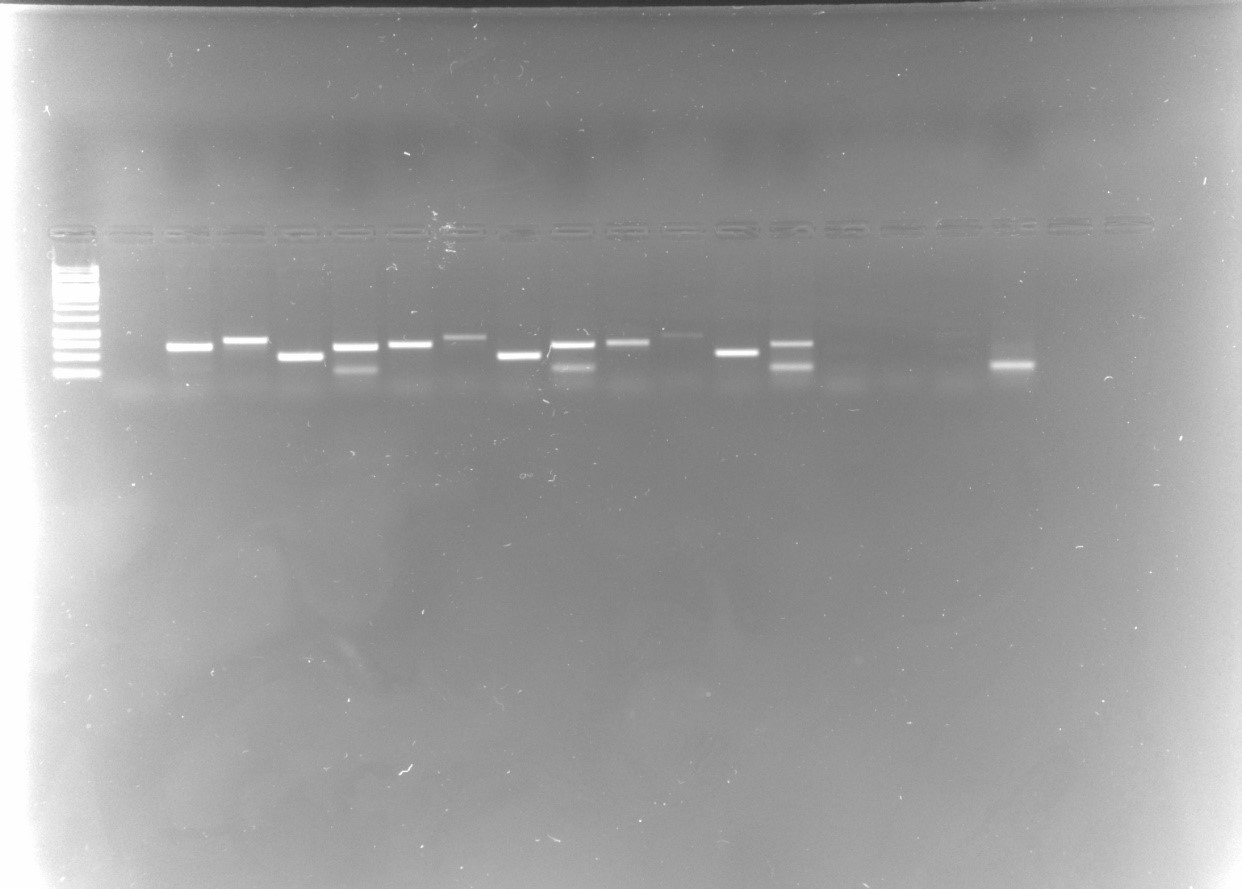


1kb ladder

Blank

20025 - A

20025 - B

20025 - C

20025 - D

50289 - A

50289 - B

50289 - C

50289 - D

70203 - A

70203 - B

70203 - C

70203 - D

70346 - A

70346 - B

70346 - C

70346 - D

Figure S2a shows 4 different sets of primers (A: 7541-7542, B: 7543-7544, C: 7545-7546, D:7547-7548) amplifying different subregions of the ovine BCO2 cDNA sequence in 4 different individuals (20025, 50289, 70203 and 70346, respectively). No fragments are amplified in the yellow fat individual (70346). The band in 70346 - D is an artefact which also is visible in the 3 other individuals, in addition to the band of expected size found in the other 3. The size marker is 1 kb GeneRuler®.

*Figure S2b*


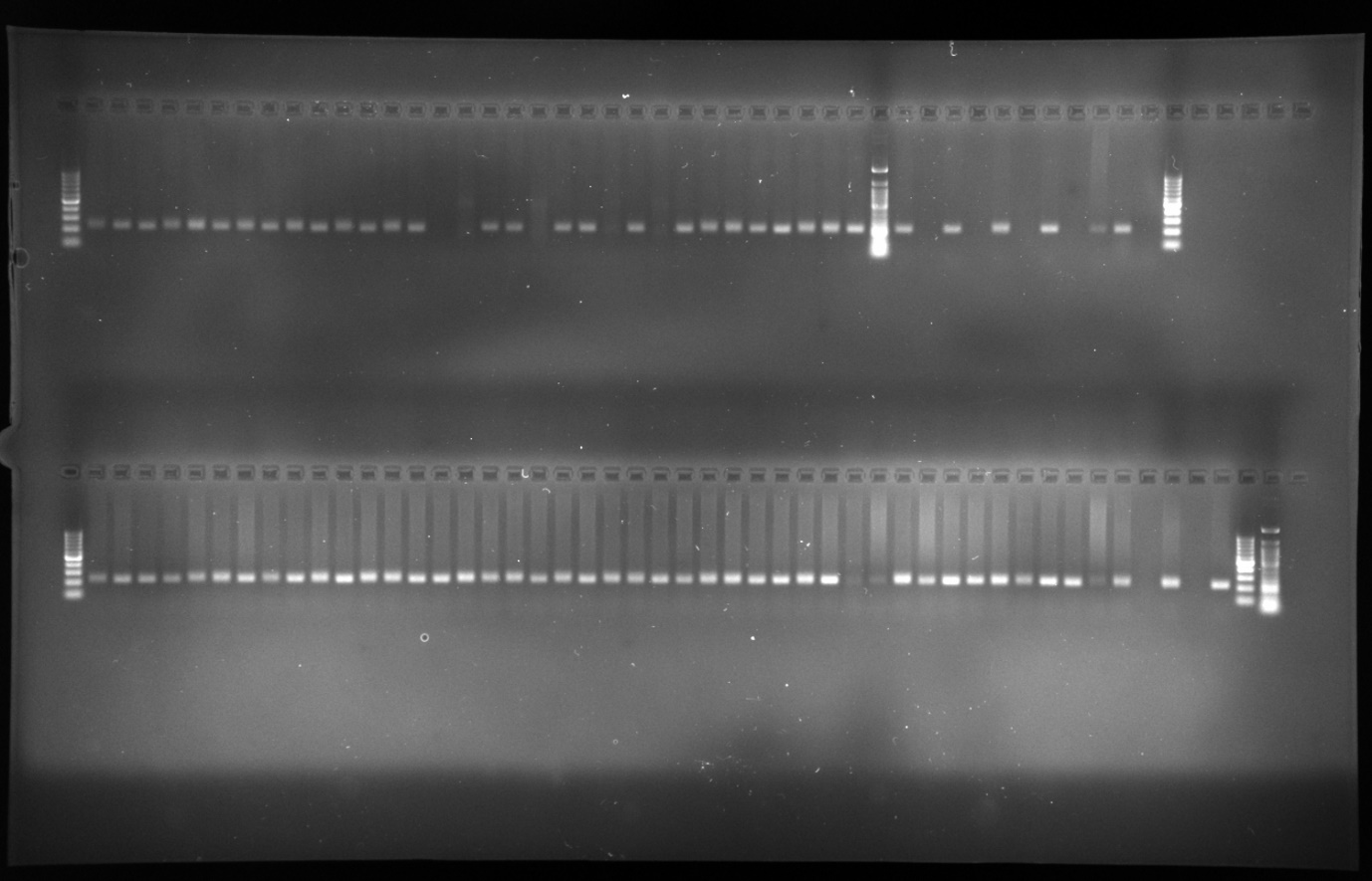


ins/ins

wt/ins

wt/wt

Figure S2b shows gel separations of PCR-products resulting from the primer pair 7552/7553. Lanes copied to the manuscript figure 2D are indicated by genotype (upper panel, lanes 24-26). Genotypes are scored as wild type (wt) or carrier of the endogenous Jaagsiekte Sheep Retrovirus (ins). The size marker to the left is 100 bp GeneRuler®.


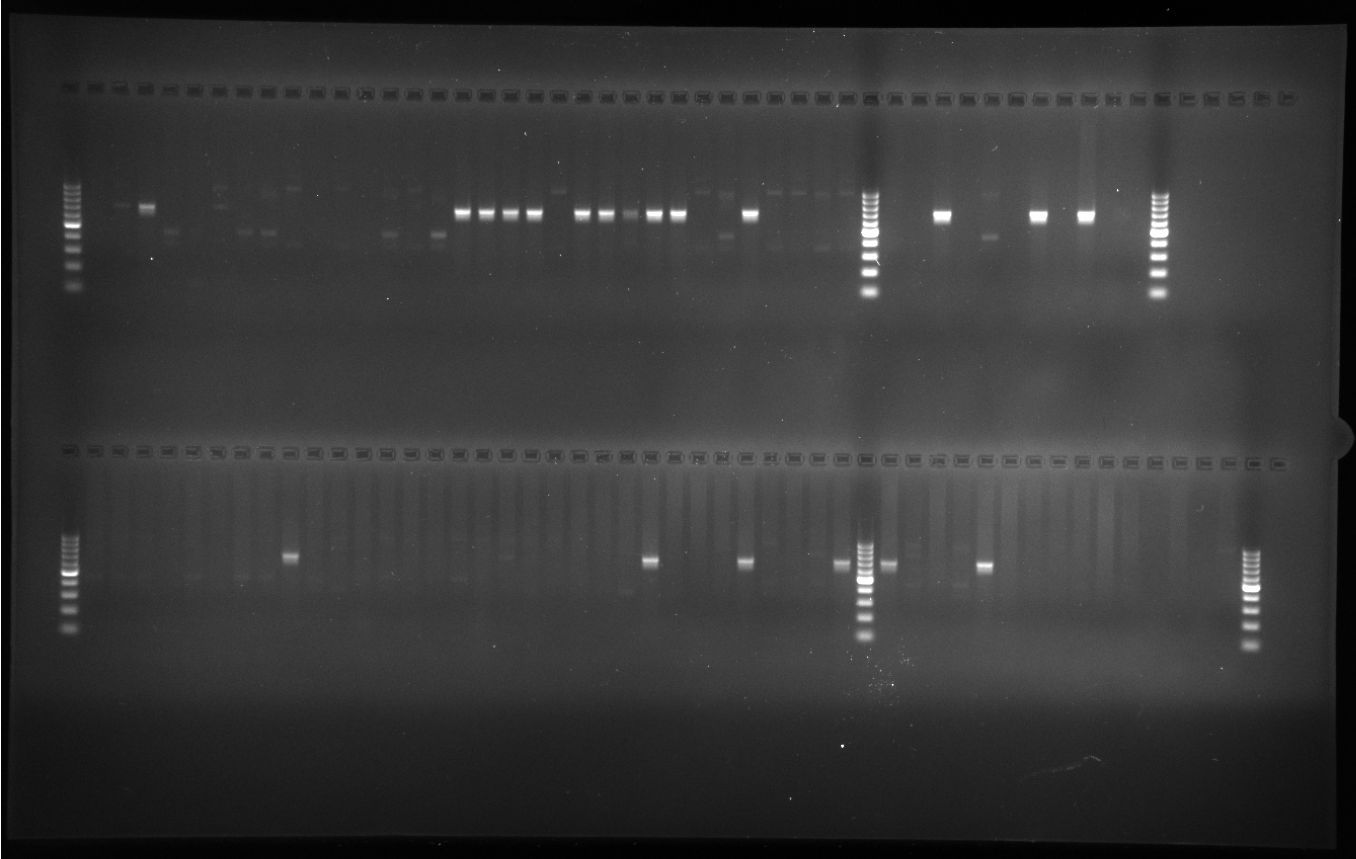


*Figure S2c*


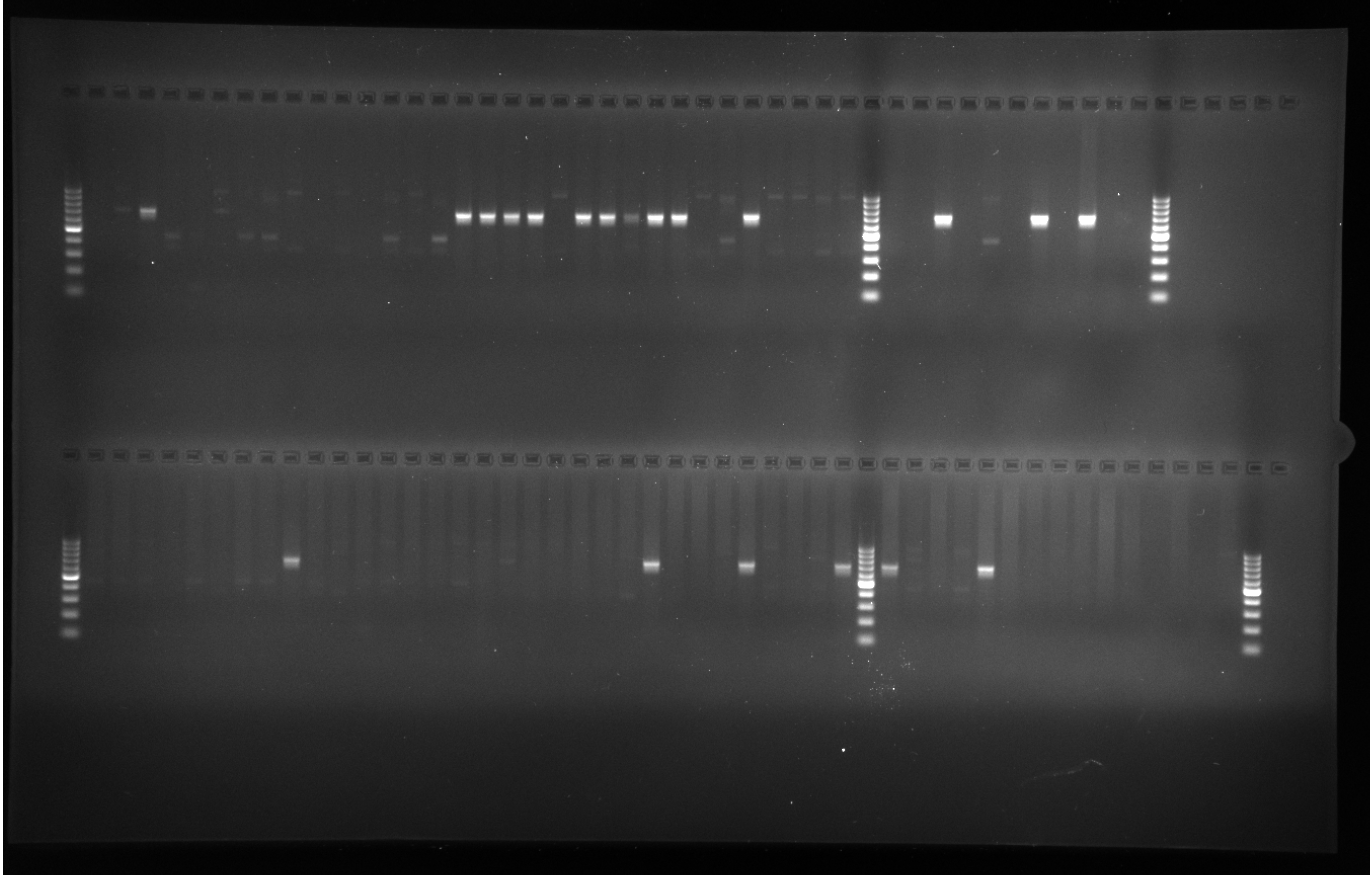


ins/ins

wt/ins

wt/wt

Figure S2c shows gel separations of PCR-products resulting from the primer pair 7554/7555. Lanes copied to the manuscript figure 2D are indicated by genotype (upper panel, lanes 24-26). Genotypes are scored as wild type (wt) or carrier of the endogenous Jaagsiekte Sheep Retrovirus (ins). The size marker to the left is 100 bp GeneRuler®.

*Figure S2d*


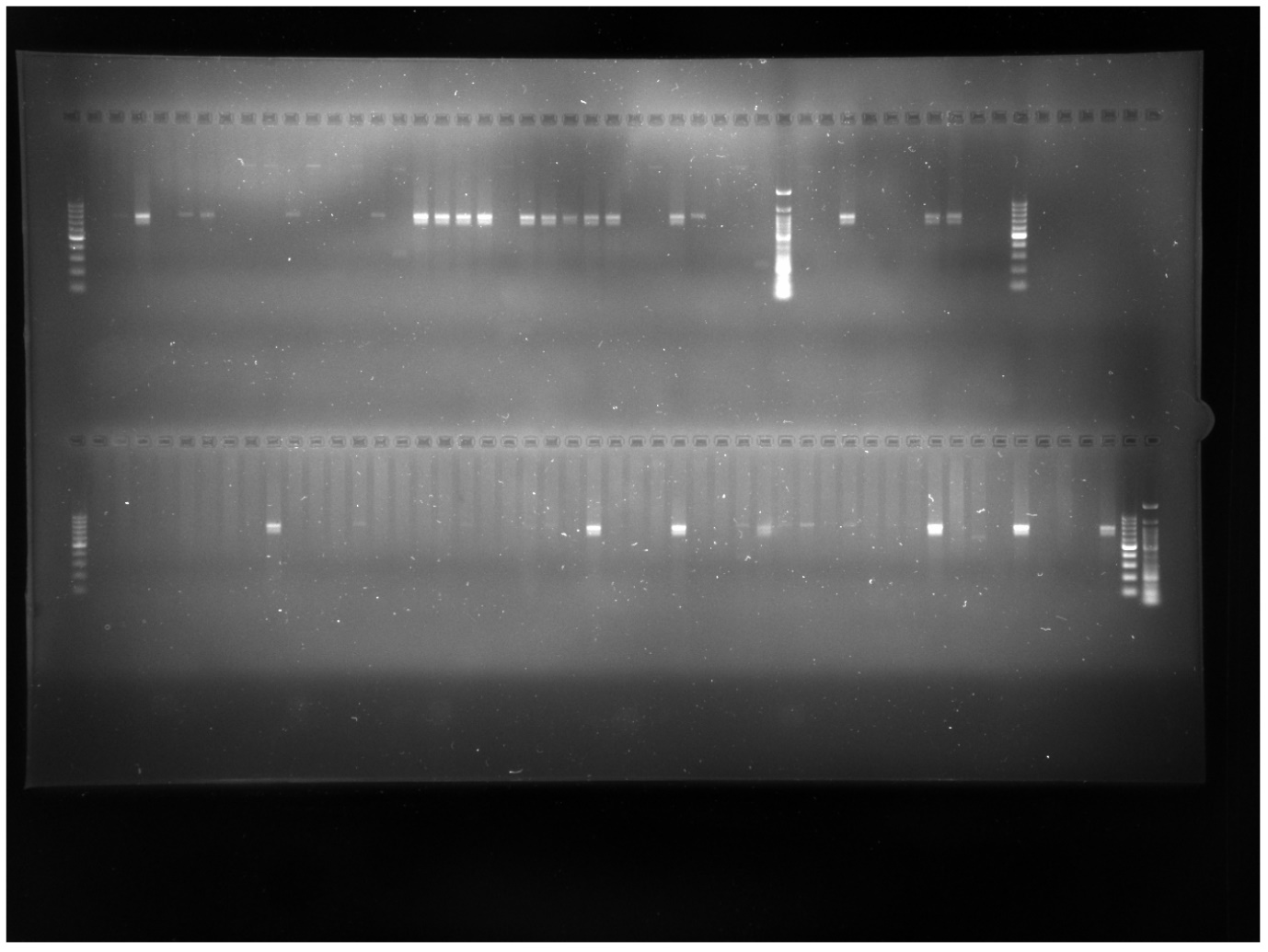


ins/ins

wt/ins

wt/wt

Figure S2d shows gel separations of PCR-products resulting from the primer pair 7556/7557. Lanes copied to the manuscript figure 2D are indicated by genotype (upper panel, lanes 24-26). Genotypes are scored as wild type (wt) or carrier of the endogenous Jaagsiekte Sheep Retrovirus (ins). The size marker to the left is 100 bp GeneRuler®.

1kb ladder

Blank

20025 - A

20025 - C

20025 - D

20025 - B

50289 - A

50289 - B

50289 - C

50289 - D

70203 - A

70203 - B

70203 - C

70203 - D

70346 - A

70346 - B

70346 - C

70346 - D
